# GADD45*β* Determines Chemoresistance and Invasive Growth of Side Population Cells of Human Embryonic Carcinoma

**DOI:** 10.4061/2010/782967

**Published:** 2010-03-14

**Authors:** Toshihiko Inowa, Keiichi Hishikawa, Yumi Matsuzaki, Takayuki Isagawa, Takumi Takeuchi, Hiroyuki Aburatani, Tadaichi Kitamura, Toshiro Fujita

**Affiliations:** ^1^Department of Urology, University of Tokyo, 7-3-1 Hongo, Bunkyo-ku, Tokyo 113-8656, Japan; ^2^Department of Clinical Renal Regeneration, Graduate School of Medicine, University of Tokyo, 7-3-1 Hongo, Bunkyo-ku, Tokyo 113-8656, Japan; ^3^Department of Internal Medicine, Division of Nephrology and Endocrinology, University of Tokyo, 7-3-1 Hongo, Bunkyo-ku, Tokyo 113-8656, Japan; ^4^Department of Physiology, Keio University School of Medicine, Shinanomachi 35, Shinjuku-ku, Tokyo, 160-8582, Japan; ^5^Genome Science Division, Research Center for Advanced Science and Technology, University of Tokyo, 7-3-1 Hongo, Bunkyo-ku, Tokyo 113-8656, Japan

## Abstract

Side population (SP) cells are an enriched population of stem, and the existence of SP cells has been reported in human cancer cell lines. In this study, we performed an SP analysis using 11 human cancer cell lines and confirmed the presence of SP cells in an embryonic carcinoma cell line, NEC8. NEC8 SP cells showed characteristics of cancer stem cells, such as high growth rate, chemoresistance and high invasiveness. To further characterize the NEC8 SP cells, we used DNA microarrays. Among 38,500 genes, we identified 12 genes that were over-expressed in SP cells and 1 gene that was over-expressed in non-SP cells. Among these 13 genes, we focused on GADD45b. GADD45b was over-expressed in non-SP cells, but the inhibition of GADD45b had no effect on non-SP cells. Paradoxically, the inhibition of GADD45b significantly reduced the viability of NEC8 SP cells. The inhibition of ABCG2, which determines the SP phenotype, had no effect on the invasiveness of NEC8 SP cells, but the inhibition of GADD45b significantly reduced invasiveness. These results suggest that GADD45b, but not ABCG2, might determine the cancer stem cell-like phenotype, such as chemoresistance and the high invasiveness of NEC8 SP cells, and might be a good therapeutic target.

## 1. Introduction

Stem cells, which have the ability to perpetuate themselves through self-renewal and differentiation, are rare in normal tissue. Several reports have shown that cancer cells also contain a small subset of cancer stem cells (CSC) with unlimited potential for self-renewal, and these cells drive tumorigenesis. CSC are characterized by the ability to generate new heterogeneous tumors and the ability to develop multidrug resistance [1, 2]. However, the characterization of CSC remains insufficient. The roots of CSC and the mechanism of tumorigenesis are considered to originate from the interaction of mutated somatic stem cells and progenitor cells [3]. CSC are more important for cancer therapy than other tumor cells because CSC might be responsible for recurrence after cancer treatment. In other words, clarifying the mechanisms responsible for the invasive growth and chemoresistance of CSC are key tasks for cancer therapy, and CSC might be a good therapeutic target. 

 Side population (SP) cells were originally reported as an enriched population of murine hematopoietic stem cells identified using Hoechst 33342 dye and FACS [4]. Recent studies have shown that this phenotype depends on the expression of ABCG2, an ATP-binding cassette (ABC) transporter [5]. SP cells have been isolated from many kinds of normal human tissues: prostate [6], limbal epithelium [7], mammary gland [8, 9], skin [10], and kidney [11–13]. Recently, SP cells have also been isolated from a variety of human cancer cell lines, including leukemia [14], neuroblastoma [15], hepatoma [16, 17], colorectal [17], thyroid [18], nasopharyngeal [19], and lung cancer [20]. Moreover, cancer SP cells are reported to have stem cell-like functions, such as chemoresistance to anticancer drugs, clonogenic ability, and tumorigenicity. In other words, cancer SP cells are promising CSC and might be a good target for cancer therapy. 

 In this study, we first tried to identify SP cells in human cancer cell lines and found a significant SP population in the embryonic carcinoma cell line NEC8. Compared to non-SP cells, the SP cells showed not only rapid growth and chemoresistance, but also rapid invasive growth. To clarify the mechanisms of the chemoresistance and invasive growth of SP cells, we performed a microarray analysis. We identified 13 genes that were differentially expressed between SP and non-SP cells. Among the 13 genes, we focused on GADD45*β*. GADD45*β* belongs to the growth arrest- and DNA damage-inducible protein family and is related to NF-kB, which is known to influence tumorigenesis, cancer cell survival, apoptosis, invasion, and metastasis [21, 22]. GADD45*β* was overexpressed in non-SP, but the knockdown of GADD45*β* paradoxically reduced the cell viability of NEC8 SP cells, but not of non-SP cells. Moreover, the invasive growth of NEC8 SP cells was reduced by the inhibition of GADD45*β*, but not by the inhibition of ABCG2, which determines the SP phenotype. Our results suggest that the CSC-like phenotype, such as resistance to anticancer drugs and invasive growth, might not only be determined by ABCG2, but also by GADD45*β*.

## 2. Materials and Methods

### 2.1. Cell Cultures

Several human cancer cell lines were used: kidney (ACHN, Caki-1, OS-RC-2, RCC10RGB), prostate (DU145, LNCap.FGC, PC3), bladder (EJ-1, RT4, T24), embryonic carcinoma (NEC8), and breast cancer (MCF-7). OS-RC-2, LNCap.FGC and NEC8 were cultured in RPMI 1640 (Sigma-Aldrich, St. Louis, http://www.sigmaaldrich.com/). RCC10 RGB was cultured in Dulbecco's Modified Eagle's Medium-low glucose (Sigma-Aldrich). PC3 was cultured in F-12 Nutrient Mixture (Ham's F-12; Gibco, Grand Island, NY, http://www.invitrogen.com/). MCF-7 was maintained in Dulbecco's modified Eagle's medium (Gibco) and the remaining cell lines were cultured in minimal essential medium (Gibco). Each medium was supplemented with 10% fetal bovine serum (Sigma-Aldrich). Only ACHN was supplemented with 1% MEM Nonessential Amino Acids Solution (Sigma). All the cells were incubated at 37°C in an atmosphere containing 5% CO_2_. ACHN, LNCap.FGC, and PC3 were obtained from Dainippon Sumitomo Pharma (Osaka, http://www.ds-pharma.co.jp/). OS-RC-2 and RCC10RGB were obtained from RIKEN (Tokyo, http://www.riken.go.jp/). DU145 and MCF-7 were obtained from the American Type Culture Collection (ATCC; Manassas, VA, http://www.atcc.org/), and the other cell lines were obtained from the Health Science Research Resources Bank (HSRRB; Tokyo, http://www.jhsf.or.jp/).

### 2.2. SP Cell Analysis and Cell Sorting

The cells were detached from the dishes with 0.05% Trypsin EDTA (Gibco) and resuspended at 2 × 10^6^ cells/mL in DMEM/F12 (Gibco) with 2% FBS. The cell suspensions were incubated with 5 *μ*g/mL Hoechst33342 (Sigma-Aldrich) for 60 minutes at 37°C. To confirm the SP population, an aliquot was stained with Hoechst33342 in the presence of 50 *μ*M of reserpine (Daiichi-sankyo, Tokyo). Both Verapamil and reserpine are commonly used for inhibiting the ABC transporter in SP analyses [23], but reserpine was more effective for SP analysis under our staining conditions. The cells were resuspended in Hank's balanced salt solution (Gibco) supplemented with 0.2% FBS containing 7AAD (BD Biosciences, San Diego) at a final concentration of 0.25 *μ*g/mL to enable the discrimination of dead cells. Cell analysis and sorting were performed using a FACS Vantage (BD Biosciences). Hoechst33342 was excited at 400 nM, and fluorescence emission was detected using a 424/44 BP and 585/42 BP optical filter for Hoechst blue and Hoechst red, respectively, and a 510-nm long-pass dichroic mirror to separate the emission wavelengths. Both Hoechst blue and red fluorescence were displayed using a linear scale. 7AAD fluorescence was measured using a 675/20 BP after excitation at 499 nM with an argon laser.

### 2.3. Drug Sensitivity Assay

Sorted NEC8 SP and non-SP cells were seeded at a density of 3 × 10^3^ cells into 96-well plates containing 100 *μ*L per well of the appropriate growth medium. The plates were then incubated at 37°C in a humidified air atmosphere containing 5% CO_2_ for 48 hours. The cells were treated with cisplatin (0.01 to 10 *μ*M) for 2 hours. After another 120 hours, the viability of the cells was measured using a colorimetric water-soluble tetrazolium salt assay (Cell Counting Kit-8, CCK-8), (Dojindo, Kumamoto, Japan, http://www.dojindo.co.jp/) according to the manufacturer's instructions, and the colorimetric analysis was detected using ARVO SX (Perkinelmer, Boston).

### 2.4. Invasion Assay

BD BioCoat Matrigel 24-well invasion chambers (BD Biosciences) were used according to the manufacturer's instructions. Cells were seeded into the upper inserts at a density of 1.5 × 10^5^ per insert in serum-free RPMI1640. A culture medium containing 10% FBS was used as a chemoattractant in the lower wells. After 48 hours, the noninvaded cells on the upper surfaces of the membranes were removed using a cotton swab. Then, the membranes were fixed with 100% methanol and stained using Giemsa dye. The invasiveness of the cells was determined by counting the number of cells that had penetrated through the pores and onto the lower side of the membrane using a microscope at a magnification of ×100. The number of cells that had migrated through the Matrigel was normalized by the mean cell count of the cells treated with NSC siRNA that were used in each invasion experiment. The invasiveness was expressed as the mean number of cells in five randomly selected fields. The percentage of invasion was determined as follows: % invasion = mean number of cells invading through the Matrigel insert membrane/mean number of cells migrating through the control insert membrane [24]. The assays was carried out as three independent experiment. 

### 2.5. RNA Extraction and Oligonucleotide Microarray Analysis

Total RNA was extracted from the NEC8 SP and non-SP cells using TRIZOL (Invitrogen, Carlsbad, CA, http://www.invitrogen.com/) according to the manufacturer's protocol. The purity of the RNA was checked using an Agilent 2100 Bioanalyzer (Agilent Technologies, Palo Alto, CA). We used the Human Genome U133A Plus2.0 arrays (Affimetrix, Santa Clara, CA), which contain almost 54,000 probe sets representing more than 47,000 transcripts including 38,500 well-characterized human genes. The preparation of the cRNA and the hybridization of the probe arrays were performed according to the protocols of the manufacturer.

### 2.6. Real-Time Quantitative PCR Analysis

Total RNA was extracted from SP and non-SP cells using an RNeasy Mini Kit (Qiagen, Valencia, CA, http://www1.qiagen.com/) according to the manufacturer's protocol. cDNA was reverse-transcribed from total RNA (20 ng) using a High Capacity cDNA Archive Kit (Applied Biosystems, Foster City, CA, https://www2.appliedbiosystems.com/) and RT reactions were performed in a GeneAmp PCR System 9700 thermal cycler (Applied Biosystems). TaqMan polymerase chain reaction (PCR) was carried out with the TaqMan Universal PCR master mix (Applied Biosystems) and TaqMan Gene expression assay (Applied Biosystems) according to the manufacturer's instructions. PCR amplification was performed using a 7500 Real-Time PCR System (Applied Biosystems). The TaqMan primer IDs for each of the analyzed genes were as follows: ABCG2, Hs00184979_g1; GADD45*β*, Hs00169587_m1; and GAPDH, Hs99999905_g1.

### 2.7. siRNA Duplexes and Transfection

All siRNA duplexes were purchased as HP Genome Wide siRNA from Qiagen and transfected using HiPerFect (Qiagen). ABCG2 siRNA (50 nM) and GADD45*β* siRNA (50 nM) were transfected 24 hours after seeding. The cells were exposed to cisplatin 48 hours after seeding.

## 3. Results

### 3.1. SP Phenotype in Human Cancer Cell Lines

**Figure 1:**
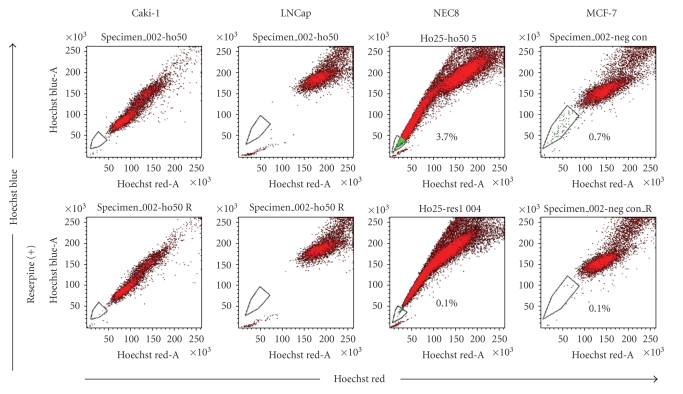
Representative FACS analysis of side population cells in human cancer cell lines. Caki1 and LNCap cells are shown as representative negative results. The SP fractions in NEC8 and MCF7 were 3.7% and 0.7%, respectively. The SP fractions disappeared in the presence of reserpine (bottom panels).

We performed a flow cytometry analysis using Hoechst 33342 dye staining (SP cell analysis) in 12 human cancer cell lines (ACHN, Caki-1, OS-RC-2, RCC10RGB, DU145, LNCap.FGC, PC3, EJ-1, RT4, T24, NEC8, and MCF7) (Figure 1). The SP gate was defined as the diminished region in the presence of reserpine, which blocked the activity of the Hoechst 33342 dye transporter. The SP fraction in MCF7 has already been reported [23], and we used MCF7 as a positive control for the gating. However, we confirmed an SP fraction only in NEC8 and MCF7, ranging from 0.7% to 3.7%, among the 12 human cancer cell lines using our Hoechst 33342 dye staining condition (60 minutes). 

### 3.2. Growth and Chemoresistance of Cancer SP

**Figure 2:**
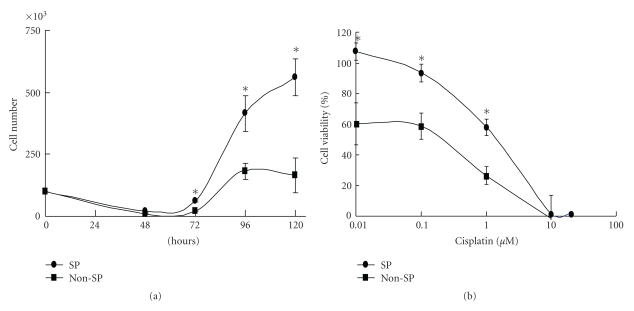
Cell growth and Cisplatin resistance. (a) Growth curve of NEC8 SP (black circles) and non-SP (black squares) cells. (b) Concentration response curve of cell viability after cisplatin treatment. Cell viability is shown as a percent of the control (no treatment). Values are the means ± SD (*n* = 8). *P < .01, significant difference versus non-SP.

Although we could not find any morphological difference in the FACS-sorted NEC8 SP cells and non-SP cells, we next compared the cell growth and chemoresistance of these 2 fractions. Although no difference was observed until 72 hours after seeding, the NEC8 SP cells showed rapid cell growth, compared to the non-SP cells, after 96 hours (Figure 2(a)). To examine the chemoresistance of NEC8 SP cells, cell viability was examined in the presence of cisplatin (0.01 *μ*M to 50 *μ*M). Compared with the non-SP cells, the SP cells showed a significantly higher resistance to cisplatin, ranging from 0.01 *μ*M to 1.0 *μ*M (Figure 2(b)).

### 3.3. Strong Invasiveness in SP Cells

**Figure 3:**
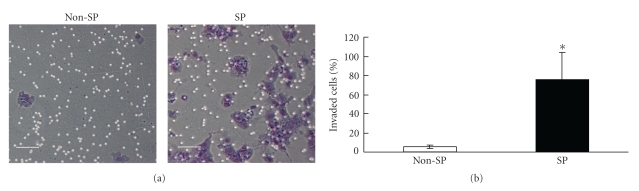
Invasion assay. (a) Representative photograph of Matrigel invasion assay. Scale bar = 50 *μ*m. (b) Comparison of invasiveness of NEC8 SP and non-SP cells. The invaded cell counts from triplicate invasion assays of three independent experiments are shown. Values are the % mean ± SD (*n* = 8). *P < .05 versus non-SP.

To clarify the difference in invasiveness between SP and non-SP cells, we performed an in vitro Matrigel invasion assay. NEC8 non-SP cells showed almost no invasion. However, NEC8 SP cells showed a level of invasiveness that was approximately 10 times greater than that of the non-SP cells (Figure 3).

### 3.4. SP Cells Regenerate Both SP and Non-SP Cells

**Figure 4:**
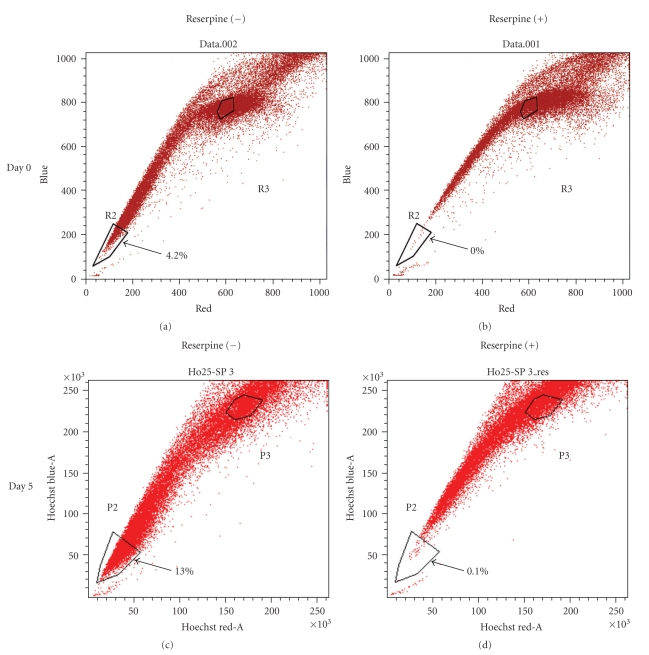
SP cells from NEC8 become SP and non-SP cells. Hoechst 33342 dye-stained NEC8 cells were sorted to extract the SP fraction (upper panel), and the SP fraction was further cultured. On day 5, the cultured cells were stained with Hoechst 33342 and reanalyzed (lower panel).

To investigate the repopulation ability of NEC8 SP cells, we cultured FACS sorted cells for five days and performed a second SP analysis again. The NEC8 SP cells generated both SP and non-SP cells, with an SP fraction size that was greater than that of the original population (4.3% versus 13.0%; Figure 4).

### 3.5. Gene Expression Profile of SP Cells Using a Microarray Analysis

**Figure 5:**
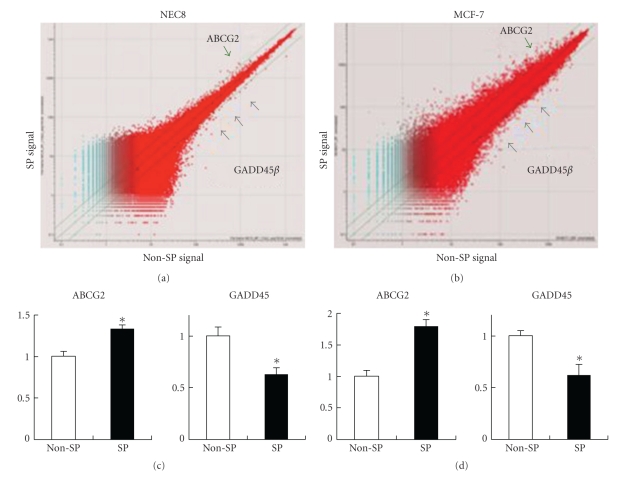
Gene expression profile of SP cells according to a microarray and real-time PCR analysis. (a and b) Scatter plot analyses of gene expression in SP versus non-SP cells from NEC8 (a) and MCF7 (b). ABCG2 is shown by the green arrow, and GADD45*β* is shown by the gray arrow. (c and d) Quantitative gene expression analysis of ABCG2 and GADD45*β* (normalized to GAPDH) using real-time PCR in NEC8 (c) and MCF7 (d). Values are the mean ± SD (*n* = 8). P* < .05 versus non-SP cells.

To investigate the gene expression profile of SP cells, we performed an oligonucleotide-based DNA microarray analysis using a GeneChip Human Genome U133 Plus 2.0 array (Affymetrix). We searched for genes with a high signal intensity (more than 100) and significant differences in expression (SP/non-SP > 1.8 or non-SP/SP > 1.8). Moreover, we selected genes that were expressed in both NEC8 SP cells and MCF7 SP cells. Among 38,500 genes, we identified 12 genes that were overexpressed in SP cells and 1 gene that was overexpressed in non-SP cells (Table 1). Unexpectedly, ABCG2, which determines the SP phenotype, was not included in this table (its SP/non-SP ratio was slightly less than 1.8), but we confirmed that ABCG2 was overexpressed in the SP cells using real-time PCR (Figures 5(c) and 5(d)). Among the 13 genes, we focused on GADD45*β*, an essential mediator of cell survival in cancer cells, that was overexpressed in non-SP cells using three different microarray probes (Figures 5(a) and 5(b)). The over-expression of GADD45*β* in non-SP cells was confirmed using real-time PCR (Figures 5(c) and 5(d)). 

### 3.6. Paradoxical Functional Role of ABCG2 and GADD45*β* in SP Cells

**Figure 6:**
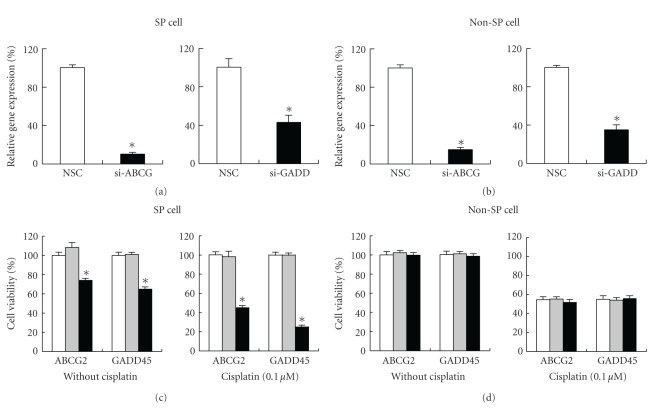
Functional roles of ABCG2 and GADD45*β* in NEC8 SP and non-SP cells. (a and b) Quantitative gene expression analysis of ABCG2 and GADD45*β* after treatment with siRNA in NEC8 SP cells (a) and non-SP cells (b). The white bar represents treatment with nonsilencing control siRNA (NSC) and the black bar represents ABCG2 siRNA and GADD45*β* siRNA. (c and d) Effect of treatment with siRNA for ABCG2 and GADD45*β* on cell viability. The white bar represents the untreated control, the gray bar represents treatment with NSC, and the black bar represents treatment with siRNA. The values are shown as the percent of the untreated control. *P < .001 versus NSC.

To clarify the functional roles of ABCG2 and GADD45*β* in NEC8 SP cells, we treated the NEC8 SP and non-SP cells with small interfering RNA (siRNA) of the genes and examined cell viability in the presence and absence of cisplatin (0.1 *μ*M). Compared to treatment with nonsilencing control siRNA, treatment with siRNA for ABCG2 and GADD45*β* significantly reduced gene expression in both NEC8 SP and non-SP cells (Figures 6(a) and 6(b)). Treatment with siRNA for ABCG2, which is overexpressed in NEC8 SP cells, significantly reduced the cell viability of NEC8 SP cells but had no effect on the non-SP cells (Figures 6(c) and 6(d)). Paradoxically, treatment with siRNA for GADD45*β*, which is overexpressed in non-SP cells, significantly reduced the cell viability of SP cells but had no effect on the non-SP cells (Figures 6(c) and 6(d)).

### 3.7. GADD45*β* Determines the Invasiveness of SP Cells, But Not the SP Phenotype

**Figure 7:**
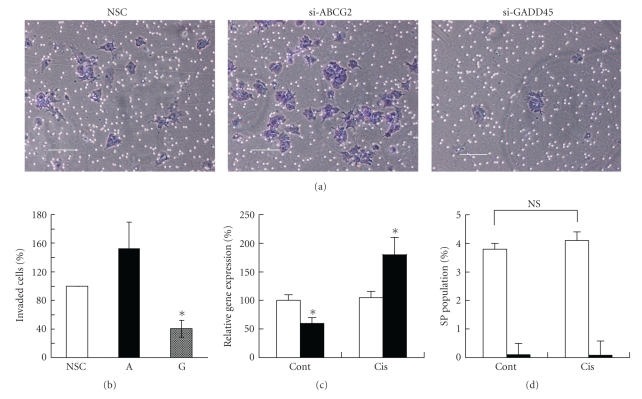
GADD45*β* determines invasion of SP cells but not SP phenotype. (a) Representative photographs of invasion of NEC8 SP cells after the inhibition of ABCG2 and GADD45*β* using siRNA. Scale bar = 50 *μ*m. (b) Comparison of invasiveness of NEC8 SP cells treated with siRNA. Values are the means of the normalized cell counts to NSC from triplicate invasion assays of three independent experiments. *P < .005 versus NSC. NSC means treatment with nonsilencing control siRNA, G means treatment with GADD45*β* siRNA, and A means ABCG2 siRNA. *P < .001 versus NSC. (c) Upregulation of GADD45*β* by treatment with a low concentration of cisplatin. NEC8 cells were incubated with vehicle (cont) or 0.01 *μ*M of cisplatin (cis) for 24 hours, and were sorted by FACS. The gene expression of GADD45*β* in SP (black bar) and non-SP (white bar) cells was evaluated using real-time PCR. The values are the mean ± SD (*n* = 8). *P < .001 versus non-SP (*n* = 8). (d) SP analysis after the upregulation of GADD45*β*. NEC8 cells were incubated with the vehicle (cont) or 0.01 *μ*M of cisplatin (cis) for 24 hours, and an SP analysis was performed in the presence (white bar) or absence (black bar) of reserpine. Values are the mean ± SD (*n* = 8). NS means not significant.

As shown in Figure 3, the SP cells showed a very strong invasiveness, compared with the non-SP cells. To clarify the mechanism of invasiveness, we treated the NEC8 SP cells with siRNA for ABCG2 and GADD45*β*. Although treatment with siRNA for ABCG2 significantly reduced gene expression (Figure 6(a)), it showed no effect on invasion (Figure 7(a) upper middle and Figure 7(b)). On the other hand, treatment with siRNA for GADD45*β* significantly prevented invasion (Figure 7(a) upper right and Figure 7(b)). As GADD45*β* belongs to the growth arrest- and DNA damage-inducible protein family, we tried to upregulate GADD45*β* in NEC8 by treating the cells with a low concentration of cisplatin (0.01 *μ*M for 24 hours). The NEC8 cells were incubated with the vehicle or cisplatin for 24 hours, and were then sorted using FACS. Treatment with a low concentration of cisplatin (0.01 *μ*M) significantly upregulated the gene expressions of GADD45*β* in NEC8 SP cells, but showed no effect on non-SP cells (Figure 7(c)). Although treatment with a low concentration of cisplatin upregulated the expression of GADD45*β* in SP cells (Figure 7(c)), an FACS analysis showed no change in the SP population (Figure 7(d)).

## 4. Discussion

The SP phenotype can be used to enrich a stem cell fraction and is determined by the presence of ABCG2 [5]. However, the physiological role of this ATP binding cassette transporter in cancer SP cells is still unclear. In this study, we confirmed the significant existence of SP cells in a human embryonic carcinoma cell line, NEC8. NEC8 SP cells showed cancer stem-like phenotypes, such as rapid growth, chemoresistance, invasive growth and self-renewal. Interestingly our results showed that GADD45*β*, but not ABCG2, might determine resistance to cisplatin and the high level of invasiveness of NEC8 SP cells. 

 ABCG2 is a member of the ABC transporters and is known to transport anticancer drugs such as doxorubicin, daunorubicin, mitoxantrone, and topotecan. GADD45*β* belongs to the growth arrest- and DNA damage-inducible protein family, and Zerbini et al. reported that the GADD45 family are essential mediators of cell survival in cancer cells, with implications for cancer chemotherapy and novel drug discovery [22]. In other words, both genes can protect cells from cell death. In fact, the inhibition of these genes using siRNA induced cell death in NEC8 SP cells. GADD45*β* was overexpressed in non-SP cells, compared with NEC8 SP cells, but the inhibition of GADD45*β* paradoxically reduced cell viability and augmented sensitivity to cisplatin in SP cells but not in non-SP cells. Moreover, a low dosage of cisplatin upregulated the expression of GADD45*β* in SP cells, but not in non-SP cells. These results suggest that the regulation and functional role of GADD45*β* differs among cell types but that GADD45*β* could be useful for curative therapy targeting cancer stem-like cells, such as SP cells. The inhibition of ABCG2, which determines the SP phenotype, had no effect on the invasion of SP cells, but that of GADD45*β* strongly inhibited it. Caution is needed in interpreting the effects of the knock-down of these two proteins. As NEC8 SP cells showed rapid growth compared with non-SP cells, the effects of siRNA might be augmented by a rapid cell cycle in NEC8 SP cells, and not by the ABCG2-determined SP phenotype itself. Although the SP population is heterogeneous, our results suggest that there might be some hierarchy of genes that determines the various phenotypes of SP cells. ABCG2 might determine the general common phenotype of SP cells, while GADD45*β* might determine part of the core functions of these cells, such as chemoresistance and invasiveness. 

 In this study, we examined 12 cancer cell lines (ACHN, Caki-1, OS-RC-2, RCC10RGB, DU145, LNCap.FGC, PC3, EJ-1, RT4, T24 and NEC8) and found SP cells only in NEC8. However, Patrawala et al. and Ning et al. have reported SP cells or stem-like populations in these cell lines [23, 25, 26]. We believe that differences in the staining conditions of their studies and ours might explain this discrepancy. Patrawala et al. [25] stained cancer cells with CD44&*α*2*β*1 or stained cells with Hoechst 33342 for 90 minutes [23]. Ning et al. [26] also stained cells with Hoechst 33342 for 90 minutes, but our incubation time was 60 minutes. Generally, a long incubation time increases the SP population. However, a long incubation time (90 minutes) somehow reduced the cell viability of NEC8, and we optimized our incubation time as 60 minutes. We believe that discussing the definite existence of small SP populations is difficult without standardizing the staining condition. Further studies are required to optimize the staining conditions, but we believe that SP analysis in cancer cells can be useful for predicting some disease characteristics that might be suitable treatment targets. 

 Zhou et al. reported that the stem-like phenotype of SP cells is determined by ABCG2 [5]. We also found a significant difference in the gene expression of ABCG2 in NEC8 SP and non-SP cells, but the difference was marginal. As the SP fraction was separated according to the functional dye-efflux capacity of ABCG2, we speculate that there might be a discrepancy between the gene expression of ABCG2 and the functional capacity of ABCG2 in NEC8 SP cells, and an alternative criteria for identifying CSC should be considered for NEC8 SP cells. No precise criteria exist for defining cancer stem cells, but several reports have proposed two major characteristics of CSC [1, 2, 27–30]: (1) an everlasting period of self-renewal (tumorigenesis), and (2) the production of progenitor cells through heterogeneous division with the ability to further differentiate. Moreover, some other reports have mentioned resistance to cancer therapy drugs as another characteristic [2, 27, 28]. In the medical literature, cancer SP cells reportedly show the ability to undergo multilineage differentiation both in vivo and in vitro [15–20]. In our study, NEC8 SP cells showed the ability to undergo self-renewal and exhibited resistance to cancer therapy drugs. To further clarify the cancer stem-like character of NEC8 SP cells, we used a xenograft model using SCID-mice, CD 133 staining [31] and sphere formation. However, we found no differences between the NEC8 SP cells and the non-SP cells. Our results were not sufficient to characterize NEC8 SP cells as cancer stem-like cells, but these cells might be a useful therapeutic target as they showed resistance to cancer therapy and are highly invasive. 

 In conclusion, our results showed that cancer cells are not homogeneous and suggest that therapeutic strategies targeting specific cell populations, such as SP cells and non-SP cells, should be considered. The CSC-like phenotype of cancer SP cells might not only be determined by ABCG2, but also by GADD45*β*. Although SP cells comprise a minor population of all cancer cells, it is very important to completely kill these cells to achieve a curative therapy, since SP cells exhibit a strong resistance to cancer therapy and are highly invasive. Taken together, our results suggest that the inhibition of both ABCG2 and GADD45*β* might be used as a new target for curative therapy without recurrence or metastasis through the targeting of cancer stem-like cells, such as SP cells. 

## Figures and Tables

**Table 1:** 

Probe Set lD	Gene symbol	Gene name	Unigene	SP/Non SP signal	SP/Non SP signal
				(NEC8)	(MCF7)
SP < non-SP					

207574_s_at	GADD45*β*	growth arrest and dna-damage-inducible, beta	Hs.110571	305.9/594.6	272.2/489.9
209304_x_at				126./249.5	121/241.6

SP > Non SP					

203002_at	AMOTL2	Kiaa0989 protein	Hs.426312	244.7/105.2	289.8/240.5
202464_s_at	PFKFB3	6-phosphofructo-2-kinase/frucrose-2, 6-biphosphatase 3	Hs.195471	343.4/164.1	502.3/177.5
232889_at	LOCI53561	Hypothetical protein loc153561	Hs.283742	277.3/152.4	455.5/92.6
232174_at	EXT1	Exostoses (multiple) 1	Hs.492618	430.2/177.2	224/68.2
201631_s_at	IER3	Immediate early response 3	Hs.76095	2249.9/960.6	3962.7/2442
221215_s_at	RIPK4	Rceptor-interacting serine-threonine kinase 4	Hs.517310	1517.6/517.8	291.9/118.8
234981_x_at	LOCI34147	Smilar to mouse 2310016a09rik gene	Hs.I92586	1382.6/660	1566.5/989.1
209501_at	CDR2	Crebellar degeneration-related protein 2	Hs.513430	355.8/187.8	307.2/123.3
202431_s_at	MYC	v-myc myclocytomatosis viral oncogene homolog (avian)	Hs.202453	1468.1/355.3	1832/599.2
220796_x_at	SLC35E1	Slute carrier family 35, member el	Hs.134074	488.5/262.8	832.3/218.3
201694_s_at	EGRI	Erly growth response 1	Hs.326035	1412/576.2	808.3/415
1562062_at	NBPF	Nuroblastoma breakpoint family, member	Hs.351620	286.6/141.1	230.4/47.4
1562063_x_at		1,3,8,9,10,11,20		320.2/126.7	301.7/106
